# Resveratrol Prevents Endothelial Cells Injury in High-Dose Interleukin-2 Therapy against Melanoma

**DOI:** 10.1371/journal.pone.0035650

**Published:** 2012-04-20

**Authors:** Hongbing Guan, Narendra P. Singh, Udai P. Singh, Prakash S. Nagarkatti, Mitzi Nagarkatti

**Affiliations:** Department of Pathology, Microbiology and Immunology, University of South Carolina School of Medicine, Columbia, South Carolina, United States of America; McMaster University, Canada

## Abstract

Immunotherapy with high-dose interleukin-2 (HDIL-2) is an effective treatment for patients with metastatic melanoma and renal cell carcinoma. However, it is accompanied by severe toxicity involving endothelial cell injury and induction of vascular leak syndrome (VLS). In this study, we found that resveratrol, a plant polyphenol with anti-inflammatory and anti-cancer properties, was able to prevent the endothelial cell injury and inhibit the development of VLS while improving the efficacy of HDIL-2 therapy in the killing of metastasized melanoma. Specifically, C57BL/6 mice were injected with B16F10 cells followed by resveratrol by gavage the next day and continued treatment with resveratrol once a day. On day 9, mice received HDIL-2. On day 12, mice were evaluated for VLS and tumor metastasis. We found that resveratrol significantly inhibited the development of VLS in lung and liver by protecting endothelial cell integrity and preventing endothelial cells from undergoing apoptosis. The metastasis and growth of the tumor in lung were significantly inhibited by HDIL-2 and HDIL-2 + resveratrol treatment. Notably, HDIL-2 + resveratrol co-treatment was more effective in inhibiting tumor metastasis and growth than HDIL-2 treatment alone. We also analyzed the immune status of Gr-1^+^CD11b^+^ myeloid-derived suppressor cells (MDSC) and FoxP3^+^CD4^+^ regulatory T cells (Treg). We found that resveratrol induced expansion and suppressive function of MDSC which inhibited the development of VLS after adoptive transfer. However, resveratrol suppressed the HDIL-2-induced expansion of Treg cells. We also found that resveratrol enhanced the susceptibility of melanoma to the cytotoxicity of IL-2-activated killer cells, and induced the expression of the tumor suppressor gene FoxO1. Our results suggested the potential use of resveratrol in HDIL-2 treatment against melanoma. We also demonstrated, for the first time, that MDSC is the dominant suppressor cell than regulatory T cell in the development of VLS.

## Introduction

High-dose interleukin-2 (HDIL-2) therapy which produces overall response rates of 15% to 23% remains an effective and long-lasting treatment for metastatic renal cell carcinoma and melanoma [1], [2]. However, it is associated with significant systemic toxicity, mainly, vascular leak syndrome (VLS), characterized by increased vascular permeability and decreased microcirculatory perfusion that causes extensive fluid retention in multiple organs and can lead to pulmonary and cardiovascular failure [3], [4], [5] Although it is dose-dependent and reversible with therapy discontinuation, there is no specific and effective treatment regimen. The endothelial cell damage is the major feature of the vascular pathology. Direct effects of IL-2 on endothelial cells [6], [7], or through induction of inflammatory cytokines such as TNF-α, IL-1 and IFN-γ [3], have been previously reported. Otherwise, the cytotoxic effects by lymphokine-activated killer cells (LAK) are the main cause of vascular injury [8], [9], [10], [11].

Immunoregulation associated with the development of IL-2-induced VLS is not known. Our lab has reported that T regulatory cells (Treg) were amplified *in vivo* by IL-2 treatment; they suppress the cytolytic killing of endothelial cells by LAK cells in the *in vitro* experiments, thereby suggesting that Treg play a role in the negative regulation on the development of HDIL-2-induced VLS [12]. Lately, functional importance of myeloid-derived suppressor cells (MDSC) in immune responses has been appreciated. MDSC are a heterogeneous population of cells consisting of myeloid progenitor cells and immature myeloid cells. They are characterized by the co-expression of the myeloid lineage differentiation molecule Gr1 and CD11b. These cells markedly expand systemically in pathological conditions, such as cancer, various infectious diseases and some autoimmune diseases [13], [14]. Accumulating evidence demonstrates that their suppressive functionality contributes to the negative regulation of immune responses including adaptive and innate immunity, such as suppressing various T-cell functions, and NK and CTL cytotoxicity [13], [14], [15], [16], [17], [18]. However, the role of MDSC in HDIL-2-induced inflammation and the development of VLS have not been elucidated.

Resveratrol, a naturally occurring polyphenol found in grapes and red wine, is widely used in animal models and possesses broad-spectrum of beneficial health effects including anti-infective, anti-inflammatory, and antioxidant properties. These interesting properties confer the cardiovascular protective capacity and ability to protect endothelial function on resveratrol [19], [20]. In cancer patients, resveratrol exhibits anticancer properties, such as suppression of tumor cell proliferation, induction of tumor cell apoptosis, increase in chemosensitization of tumor cells, and exerts chemopreventive effects [21], [22]. Our lab has reported that resveratrol suppresses tumor growth by inducing apoptosis in tumor cells through aryl hydrocarbon receptor (AhR) and by reciprocal regulation of SIRT1 and NF-κB signaling [23]. For melanoma, experiments showed that resveratrol can enhance chemical cytotoxicity to the tumor, and suppress tumor growth by inducing cell-cycle interruption and apoptosis [24], [25]. Nevertheless, effects of resveratrol on the development of VLS in HDIL-2 therapy against melanoma have not been studied.

**Figure 1:**
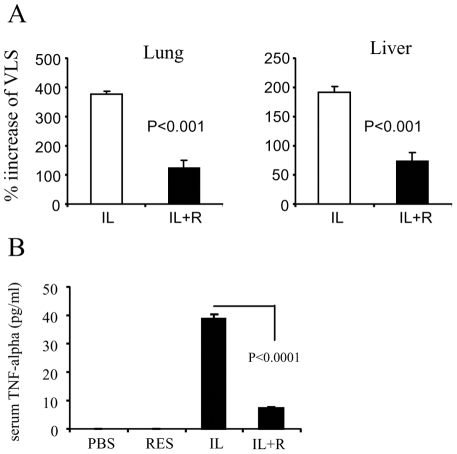
Decreased VLS in resveratrol-treated non-tumor mice. Non-tumor bearing mice were given HDIL-2 and/or resveratrol. The vascular leak in the lungs and liver as well as TNF-α in the serum were measured. A. VLS in lung. B. VLS in liver. C. The TNF-α level in the serum was measured by sandwich ELISA.

In this report, we studied the role of resveratrol in the development of VLS in B16F10 melanoma-bearing mice and revealed its role in protection of the endothelium and suppression of metastasis. We also discovered a novel functional characteristic of resveratrol in induction of suppressive activity of MDSC and expression of FoxO1 transcripts in context of suppressing the development of VLS and the growth and metastasis of B16F10 melanoma.

## Materials and Methods

### Ethics statement, mice and recombinant IL-2

Female C57BL/6 (6–8 wk of age) mice were purchased from NIH. All animals were housed in University of South Carolina Animal Facility. All animal procedures were performed according to NIH guidelines under protocols approved by the Institutional Animal Care and Use Committee of the University of South Carolina. Recombinant IL-2 was provided by the NCI Biological Resources Branch (Rockville, MD). All antibodies were purchased from Biolegend. ^3^H-thymidine and ^51^NaCr0_4_ were purchased from MP biomedical. Resveratrol was purchased from Sigma or Supelco (Bellefonte, PA).

### Induction and quantification of VLS

VLS was induced by IL-2 according to the established method from our lab [12], [26], [27], [28]. On day 0, groups of 4–5 mice were injected intraperitoneally with 75,000 units of IL-2 or PBS as a control, 3 times a day for 3 consecutive days. On day 4, the mice received one injection and 2 h later were injected intravenously with 0.1 ml of 1% Evan's blue in PBS. After 2 h, the mice were exsanguinated under anesthesia, and the hearts were perfused with heparin-PBS until lungs and livers were blanched. The lungs and livers were harvested and placed in formamide at 37°C overnight. The Evan's blue in the organs was quantified by measuring the absorbance of the supernatants at 650 nm with a spectrophotometer. For administration of resveratrol, mice were administered resveratrol (100 mg/kg body weight) on day -1 by gavage, and thereafter once a day 1 h before the first IL-2 injection during day 0 to day 4. The VLS seen in IL-2-treated mice was expressed as percentage of increase in extravasation of Evan's blue when compared with that of the PBS-treated controls and was calculated as: [(OD650 in the organs of IL-2-treated mice)-OD650 in the organs of PBS-treated controls)]/(OD650 in the organs of PBS-treated controls) ×100. Each mouse was individually analyzed for vascular leak, and data from 4–5 mice were expressed as mean ±SEM.

**Figure 2:**
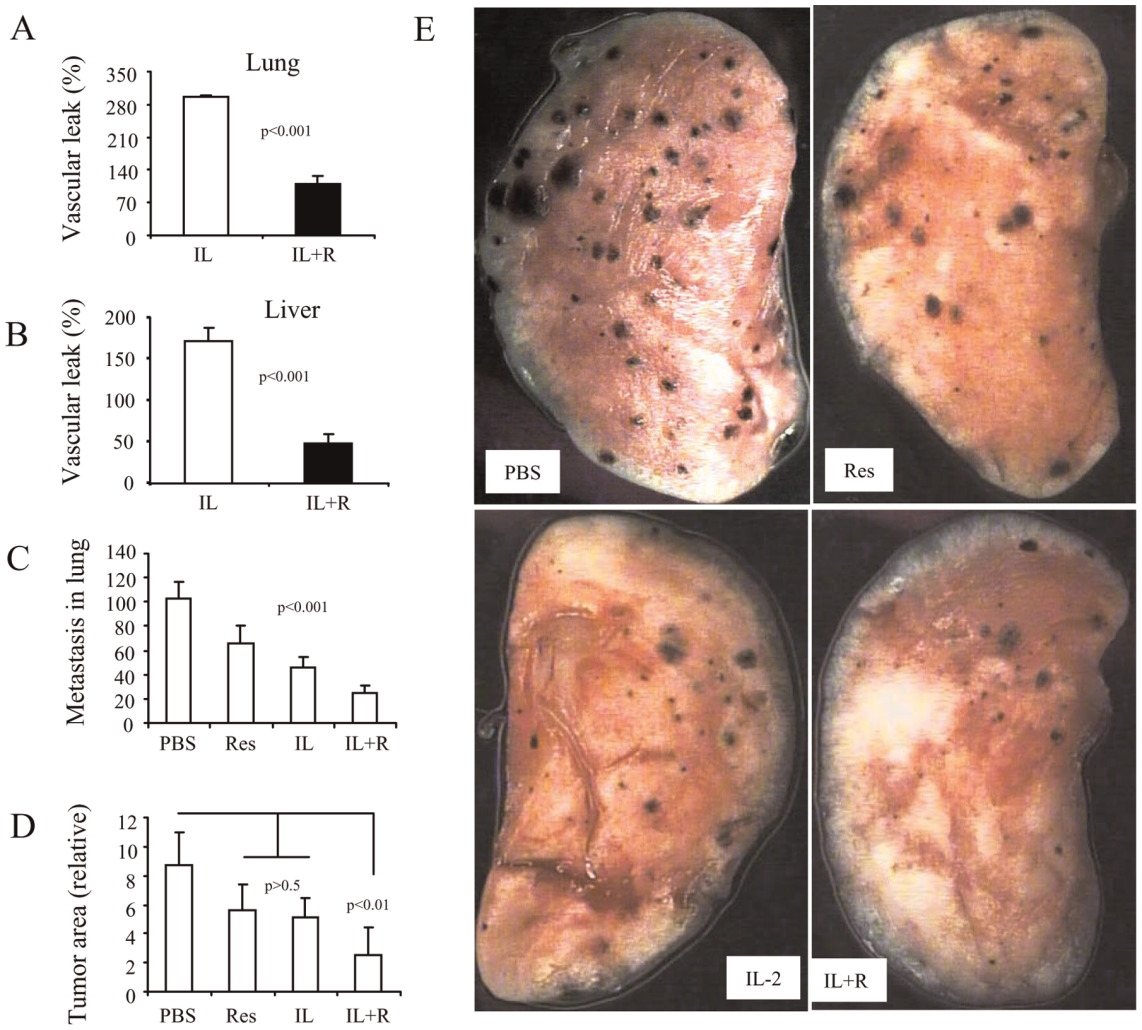
Decreased VLS and increased tumor regression in resveratrol-treated melanoma-bearing mice. C57BL/6 mice were injected *i.v.* with B16F10 melanoma cells (5×10^5^ cells/mouse) and adminsitered resveratrol as described in Fig. 1. On day 9, mice were treated with IL-2 as in Fig. 1. The mice were sacrificed for evaluation of VLS on day 12. The numbers of black metastatic nodules on the lung surfaces were counted under a microdissecting microscope. The area of each nodule was measured with software Image Pro. The values are shown as mean ±SEM. A. VLS in lung. B. VLS in liver. C. Enumeration of lung metastatic nodules. D. Area measurement of lung metastatic nodules. E. Histopathology of lung metastasis on the surface of one lobe.

### Tumor cell implantation, HDIL-2 and resveratrol treatment

Mice were injected with 5×10^5^ B16F10 cells intravenously or implanted with 2×10^5^ B16F10 cells subcutaneously on day 0. The B16F10 melanoma cell line was purchased from American Type Culture Collection (Manassas, VA) and maintained as recommended by the supplier [26]. We started to treat mice with resveratrol on day 1 and induce VLS with HDIL-2 on day 9 as described above. On day 12, mice were sacrificed to evaluate VLS and tumor metastasis in lungs. Black nodules were counted under a dissecting microscope on the surface of the lung specimens. These nodules exhibited characteristic histological features of metastatic melanomas (data not shown). The area of each nodule was measured with software Image Pro. The area of each nodule was normalized to the whole area of the same lobe and multiplied by 1000. The average area of the normalized measurement was present. For the primary tumors in the skin, they were resected and weighed, and then used for the total RNA extraction.

**Figure 3:**
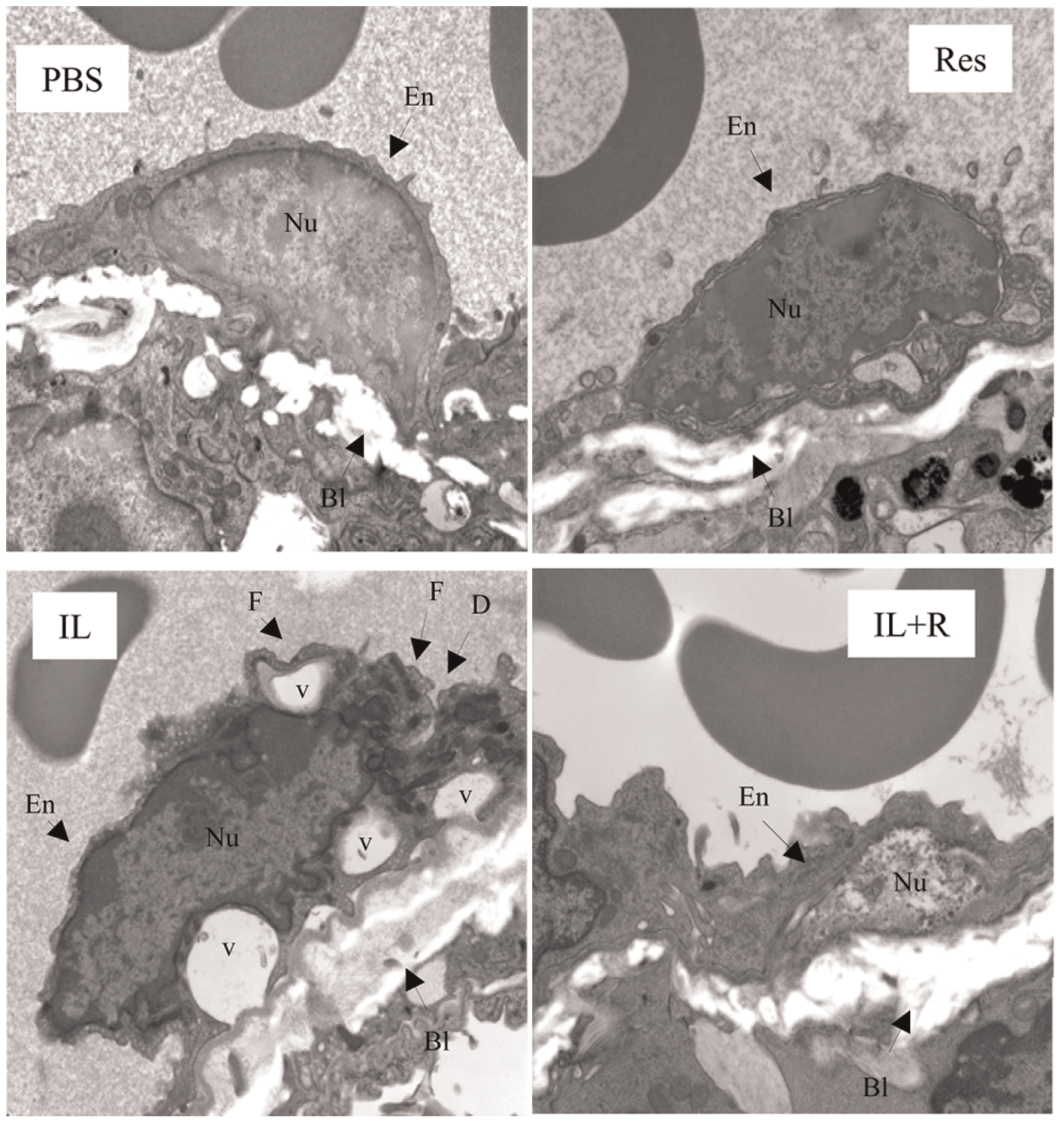
Resveratrol protects endothelial integrity. The ultrastructural studies showed resveratrol protects endothelial cells. *Panels 1* and *2* show PBS- or resveratrol-treated mice, respectively. The endothelial cell (En) lining the blood vessel is firmly anchored to the basal lamina (BL) with good integrity, thereby showing normal morphology. The cell has an intact nucleus (Nu) and is closely opposed to the intact basal lamina. Three RBCs lie within the blood vessel lumen. *Panel 3* shows IL-2-treated mice with significant damage to the endothelial cells. Cell membrane is folding (F) and discontinuous (D). Vacuolation (V) is found under the folded cell membrane and within the cytoplasm. In addition, some endothelial cells have lost the normal morphology, with only extended cell membrane remnants remaining. Cellular debris from former endothelial cells can be found in the blood vessel lumen. An RBC fills the lumen. *Panel 4* shows IL-2+resveratrol-treated mice with normal endothelial cell morphology. The cell membrane and cytoplasmic contents are well defined and closely adhere to the basal lamina. The intact nucleus is closely opposed to the intact basal lamina Three RBC fill the lumen. Original magnification, ×20,000.

### Flow cytometry studies of MDSC and Treg

For flow cytometry analysis, splenocytes were prepared. Red blood cells were lysed using red blood cell lysing buffer (Sigma). Lung and liver infiltrating inflammatory cells were isolated using Percoll gradient centrifugation (detailed below). Cells were re-suspended in staining buffer (PBS containing 2 mM EDTA and 2% fetal bovine serum) and pre-incubated for 10 min at room temperature with purified anti-CD16/32 antibody to block non-specific binding of antibodies to Fcγ receptors. For analysis of MDSC, cells were then incubated with FITC-conjugated anti-mouse CD11b and PE-conjugated anti-mouse Gr-1 mAbs in the staining buffer for 30 min at 4°C. For analysis of Treg, cells were incubated with FITC-conjugated anti-mouse CD4 mAb in the staining for 30 min at 4°C, then fixed and permeabilized with FoxP3 fix/perm buffer at room temperature in the dark for 20 min. The cells were washed once in FoxP3 perm buffer and continued to stay in FoxP3 perm buffer at room temperature for 15 min. Cells were spun down and re-suspended in 100 μl fresh FoxP3 perm buffer. Cells were then incubated with PE-conjugated anti-mouse FoxP3 mAb at room temperature in the dark for 30 min. After incubation with the mAbs, cells were washed twice in the staining buffer and re-suspended in the staining buffer. The staining was analyzed with a CXP500 flow cytometer with post-acquisition data analysis using CXP500 software (Beckman Coulter). All antibodies were purchased from Biolegend.

**Figure 4:**
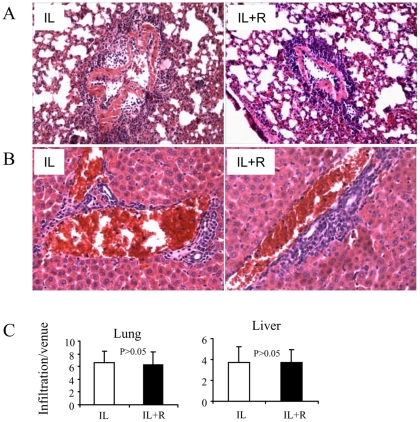
Histological studies of the lung and the liver for inflammatory cell infiltration. Lungs and livers from VLS mice were harvested and preserved in 10% formalin solution. Sections were stained with hematoxylin and eosin. The level of perivascular infiltration was determined by counting the number of cells infiltrating around a venule. The data depicts the mean ±SEM of sections from four individual mice. A. The level of perivascular infiltration in lungs. B. The level of perivascular infiltration in livers. C. Statistic analysis of the infiltration depicts p>0.05 between IL-2 and IL-2+Resveratrol groups. Original magnification, ×200.

**Figure 5:**
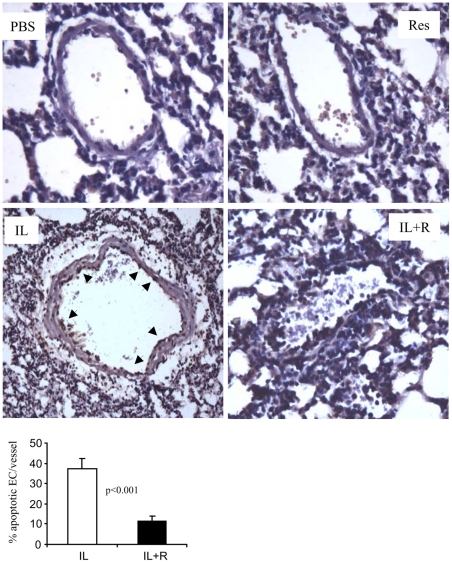
Resveratrol protects endothelial cells from apoptosis. Lungs from PBS, resveratrol, IL-2 or IL-2+resveratrol-treated mice were examined for apoptosis with TUNEL assay as described in *Materials and Methods*. Apoptotic cells are depicted by brown stained nuclei (arrow). The histogram shows the quantification of apoptotic cells. Original magnification, ×400.

**Figure 6:**
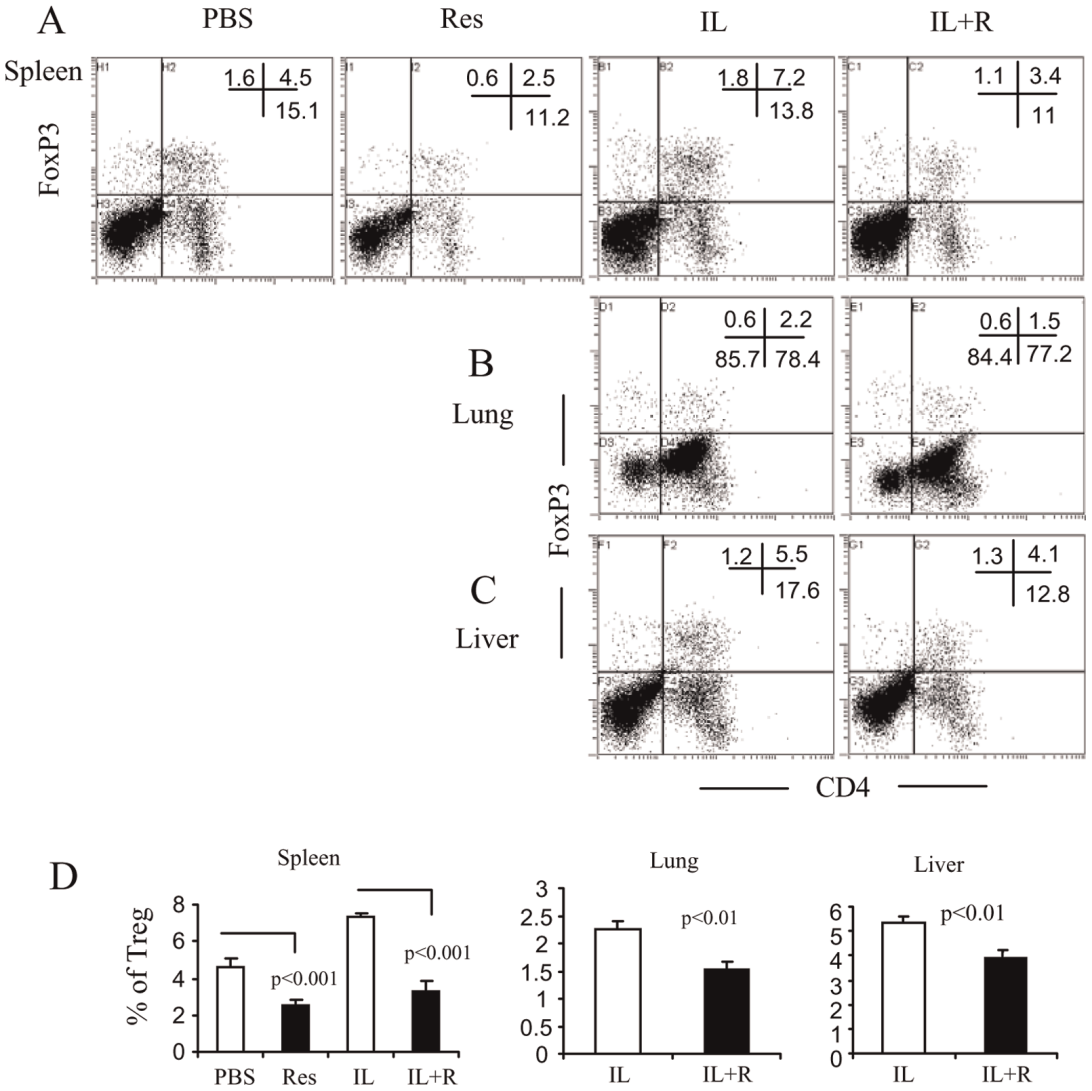
Resveratrol inhibits IL-2-induced expansion of Treg. Splenocytes and infiltrating cells were stained with FITC-conjugated anti-CD4 mAb, fixed and permeabilized and incubated with PE-conjugated anti-FoxP3 mAb as described in Materials and Methods. The stained cells were analyzed by flow cytometry. The representative dot plots from spleen (A), lung (B), and liver (C) and the statistical analysis of the percentage mean ±SEM of Treg (D) are shown.

**Figure 7:**
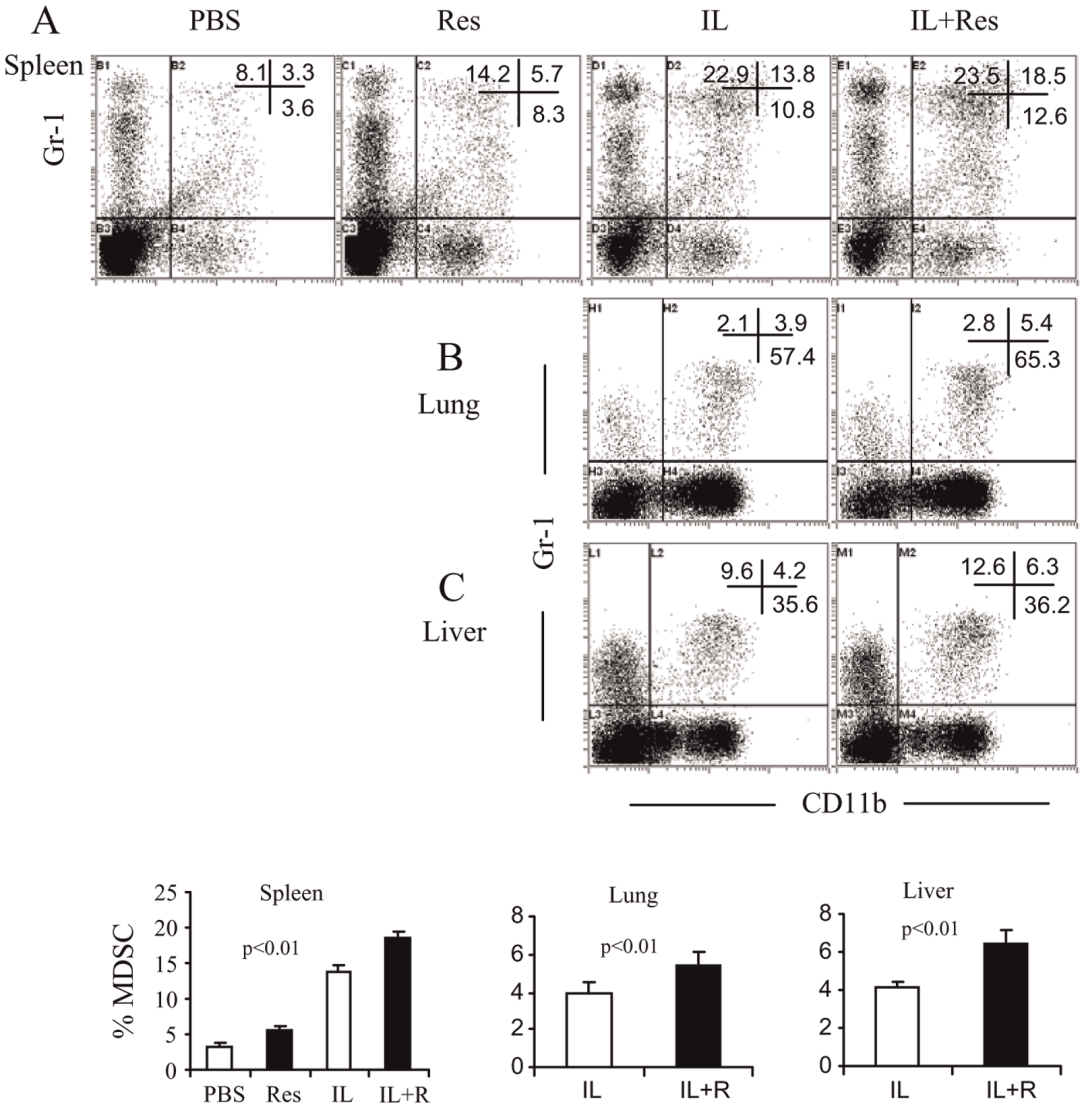
Expansion of MDSC by IL-2 and/or resveratrol treatment. Splenocytes and the infiltrating cells were stained with FITC-conjugated anti-CD11b and PE-conjugated anti-Gr-1 mAbs. The stained cells were analyzed by flow cytometry. The representative dot plots from spleen (A), lung (B), and liver (C) and the statistical analysis of the percentage mean ±SEM of MDSC (D) were shown.

**Figure 8:**
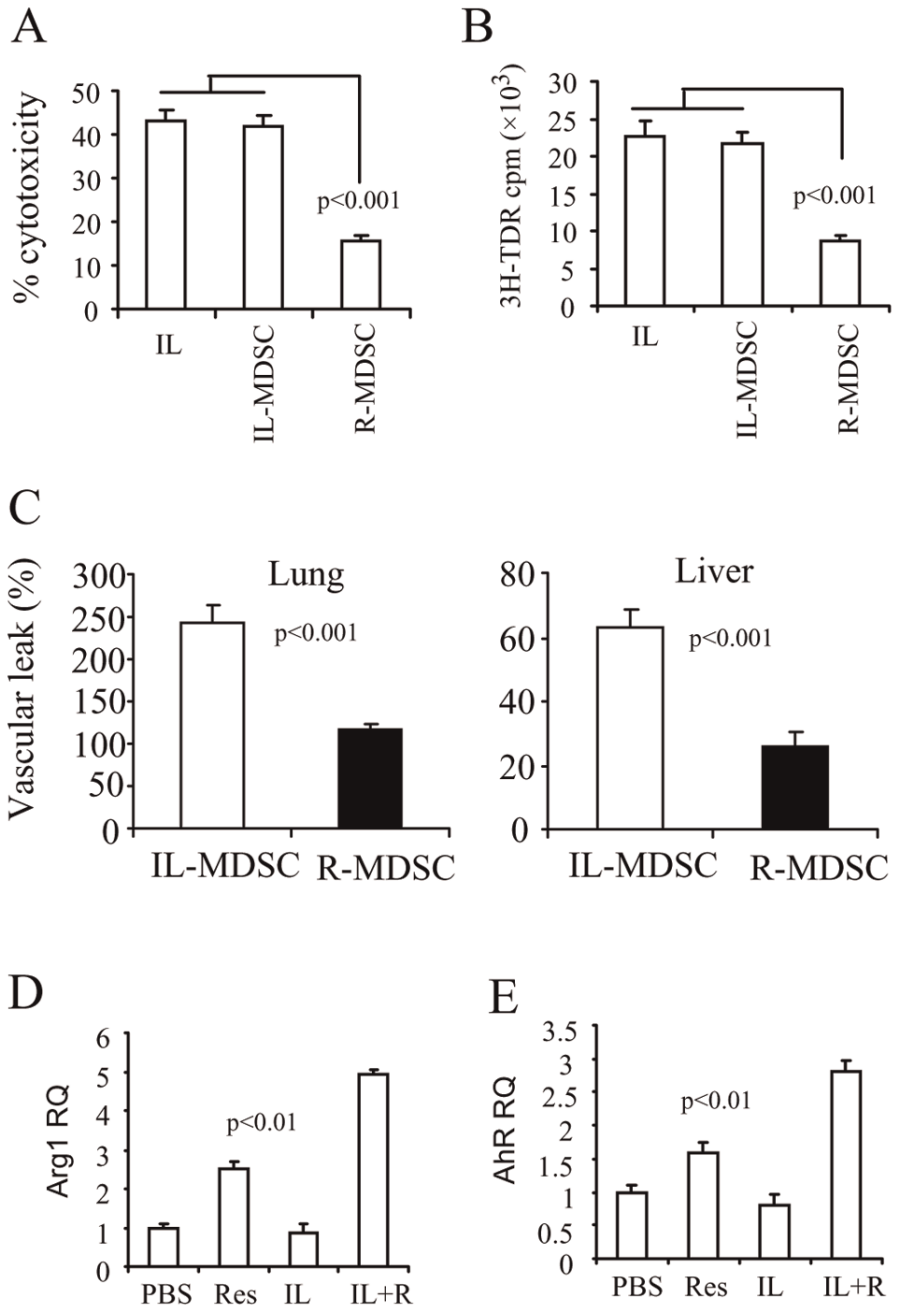
Analysis of the functional characteristics of MDSC. *Panel A:* MDSC were isolated from IL-2 and IL-2+resveratrol treatment (labeled as IL-MDSC and R-MDSC respectively). LAK cells from IL-2 treated mice were isolated and incubated with MDSC for 2 h, and then used as effectors in the cytotoxicity assay against EC cell targets. *Panel B*: ^3^H-thymidine incorporation assay to measure the proliferation of the LAK cells in the panel A. *Panel C:* 1×10^6^ MDSC were intravenously transferred to naive mice. VLS was then induced in the recipient mice. *Panel D & E:* Total RNA was extracted from the MDSC with the respective treatment. Expression of Arg1 (D) and AhR (E) were detected by QPCR. The relative expression was normalized to the endogenous 18S.

**Figure 9:**
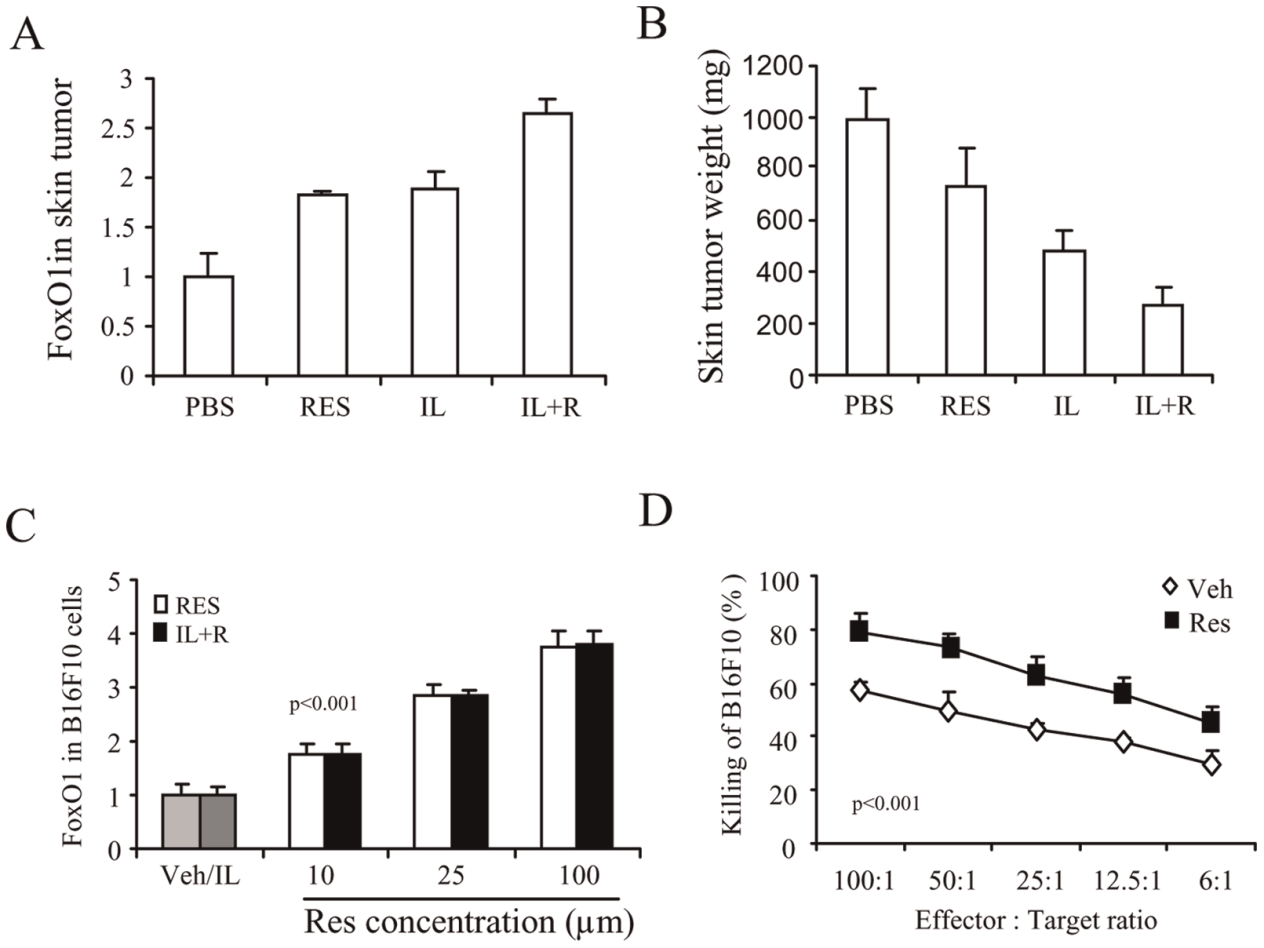
Resveratrol enhances the expression of FoxO1 and the cytolytic susceptibility of melanoma. *Panel A and B:* C57BL/6 mice were implanted *s.c.* with B16F10 melanoma cells (2×10^5^ cells/mouse) and orally administered with resveratrol. On day 9, mice were treated with IL-2 as described in Fig. 1. The mice were sacrificed for evaluation of VLS on day 12. The primary tumors in the skin were resected and weighed. Total RNA was extracted. Expression of FoxO1 was detected by QPCR (A). The relative expression was normalized to the endogenous 18S. The mean ±SEM of weight is shown (B). *Panel C:* B16F10 cells were cultured with vehicle (Veh), IL-2 (IL) (1000 units/ml), or resveratrol at the indicated dose for 24 h. Total RNA was extracted. Expression of FoxO1 was detected by QPCR. *Panel D:* LAK cells were generated by culturing splenocytes from naïve mice with IL-2 (1000 units/ml) for 48 h. B16F10 cells were incubated with resveratrol (25 µM) for 2 h and labeled with ^51^Cr. The labeled B16F10 cells were then added to the LAK cells. After 4 h, the release of ^51^Cr was measured and percent cytotoxicity was calculated. Statistical analysis was performed at each effector to target ratio and showed p<0.001.

### MDSC sorting and adoptive transfer

MDSC were sorted by two steps. First, MDSC cells were enriched from splenocytes of VLS mice by depletion of other cells (CD4+, CD8+, CD90.2+, B220+, NK1.1+, F4/80+, CD11C+, CD117+) with magnetic beads, and then, CD11b^+^Gr-1^+^ double positive cells were sorted by a flow cytometer. Briefly, splenocytes at a density of 1×10^8^ cells/ml in the staining buffer were pre-incubated for 10 min with purified anti-CD16/32 antibody to block non-specific binding of antibodies to Fcγ receptors, and then incubated with biotin-conjugated anti-mouse CD4, CD8, CD90.2, B220, NK1.1, F4/80, CD11c, and CD117 mAbs for 15 min at room temperature. Cells were washed once with and re-suspended in the staining buffer at the same cell density. Cells were then incubated with EasySep biotin cocktail (Stemcell) at room temperature for 15 min followed by incubation with EasySep magnetic particles (Stemcell) at room temperature for 10 min. Non-magnetic binding cells were collected, and further incubated with FITC-conjugated anti-mouse CD11b and PE-conjugated anti-mouse Gr-1 mAbs for 30 min at 4°C. Cells were washed twice with and re-suspended in the staining buffer. CD11b and Gr-1 double positive cells were then sorted by a FACSAria sorter. The purity was >98%. For adoptive transfer, 1×10^6^ cells were transferred per recipient mouse intravenously.

### Generation of LAK cells and ^51^Cr release assay

LAK cells were prepared as previously described [12], [26], [27], [28]. For generation of LAK cells *in vitro*, splenocytes from naïve mice were cultured for 48 h with 1000 units/ml IL-2 in RPMI containing 10% fetal bovine serum, 10 mM HEPES, 1 mM sodium pyruvate, 2 mM L-glutamine, 0.1 mM non-essential amino acid, 100 units/ml penicillin, 100 μg streptomycin, and 50 µM β-mercaptoethanol. Viable cells were purified by density gradient centrifugation using NycoPrep (Cedarlane, Burlington, NC). Such cells will be referred to as LAK cells. For generation of LAK cells *in vivo*, mice were treated with IL-2 as described above. On day 4, splenocytes were prepared and viable cells were purified using NycoPrep and referred to as LAK cells. Cytotoxicity of LAK cells was tested against B16F10 tumor or TME endothelial cells using 4 h ^51^Cr-release assay. Briefly, 1×10^6^ target cells were labeled with 100 µCi of ^51^NaCrO_4_ (MP Biomedicals) at 37°C for 1 h, washed three times in RPMI 1640 culture medium, and adjusted to 1×10^5^ cells/ml. In brief, 1×10^4^ target cells were plated into 96-well U-bottom plates with varying numbers of LAK cells depending on the respective E:T cell ratios. In these experiments, the LAK cells were defined as the effector cells (E) that mediate lysis of the ^51^NaCrO_4_-labeled endothelial cells or tumor cells, which were defined as the target cells (T). In some groups, target cells were incubated with resveratrol (25 µM) for 2 h at 37°C before they were added into the plates. For analysis of MDSC suppression, LAK cells were incubated with MDSC for 2 h before adding to the plates. Spontaneous release of ^51^NaCrO_4_ was determined by culturing the target cells alone, and the maximum release of ^51^NaCrO_4_ was determined by incubating the target cells with 1% SDS. The supernatants were harvested after 4 h culture, and the radioactivity was measured using a Microbeta counter (PerkinElmer). Percentage of killing efficiency of LAK cells was calculated as percent cytotoxicity  =  [(sample cpm − spontaneous release cpm)/(maximum release cpm − spontaneous release cpm)] ×100. The data were represented as mean ±SEM. Each group had four wells.

### Isolation of T cells and infiltrating inflammatory cells

To isolate T cells from spleen, splenocytes were stained with anti-CD3 Abs, followed by magnetic beads isolation. For isolation of infiltrating mononuclear cells (MNCs) from lung or liver, mice were perfused. Lung or liver was minced and passed through 100 μm filter. Single-cell suspensions were subject to Percoll gradient (70%/30%) centrifugation.

### TUNEL assay of cell apoptosis

Cell apoptosis was evaluated with conventional TUNEL assay using DeadEnd Colorimetric TUNEL System from Promega by following the manufacturer's instructions. Numbers of TUNEL-positive stained endothelial cells were counted in 5 vessels of each slide and the data were expressed as mean ±SE of apoptotic cells per vessel.

### Histological Analysis of cell infiltration and measurement of serum TNF-α

The lungs and livers were fixed in 10% formalin solution. The organs were embedded in paraffin, sectioned, and stained with hematoxylin and eosin. Perivascular infiltration was scaled by counting the number of lymphocytes infiltrating around the vessel and averaging the minimum and maximum range for each group. Five samples per mouse were analyzed, and a minimum of four mice were included. Sera were prepared for detection of TNF-α by sandwich ELISA using TNF-α ELISA kit (Biolegend).

### Electron microscopic studies of vascular injury

Tissue samples were fixed in 5% glutaldehyde/4.4% formaldehyde/2.75% picric acid in 0.05 M sodium cacodylate buffer, pH 7.4, washed in a sodium cacodylate buffer, postfixed in osmium tetroxide, embedded in Polybed 812 resin (Polysciences, Warrington, PA) and studied with an electron microscope.

### [^3^H]-thymidine incorporation assay

LAK cells generated from IL-2 treated mice were cultured in complete RPMI 1640 medium in a 96-well round bottom plate. [^3^H]-thymidine (1 µCi/well) was added to the culture. In some groups, LAK cells were incubated with MDSC at 10∶1 ratio (LAK:MDSC) for 2 h before adding [^3^H]-thymidine. At 16 h, cultures were harvested using a cell harvester and the radioactivity incorporated in the cells was measured in a beta counter (Microbeta counter, Perkin Elmer).

### Quantitative real-time PCR (QPCR)

Total RNAs were prepared and reversely transcribed to cDNA using miRNeasy Mini Kit and miScript Reverse Transcription Kit (Qiagen). FoxO1, arginase 1 (Arg1), aryl hydrocarbon receptor (AhR), and 18S RNA (18S) were amplified using miScript SYBR Green PCR kit (Qiagen) in a StepOnePlus PCR amplifier (ABI). The relative gene expression level was normalized to the endogenous control 18S. The primer sequences were listed below. FoxO1 F: TGT TAG GCC CAC CAC ACG AGT TTA; FoxO1 R: GGT GAA AGC AAA GGG CTC CAA TGT; Arg1 F: ACG GCA GTG GCT TTA ATT GGC; Arg1 R: TCT GTC TGC TTT GCT GTG ATG CC; NOS2 F: TGC CAC CAA CAA TGG CAA CAT C; AhR R: TTG AAG TCA ACC TCA CCA GCA GC; 18S F: AAA GGA ATT GAC GGA AGG GCA CC; 18S R: AAC TAA GAA CGG CCA TGC ACC AC.

### Statistical analysis

Data were presented as mean ±SEM and analyzed for significance using non-paired Student's t-test. In case of multiple testing, One-way ANOVA test followed by Newman-Keuls Multiple Comparision Test was applied using Graphpad Prism 5 software. Comparisons were considered significant at p≤0.05.

## Results

### Resveratrol prevents VLS while maintaining the effectiveness of HDIL-2 treatment

HDIL-2 has been shown to have significant efficacy against advanced stages of melanoma. However, HDIL-2 treatment is accompanied by significant toxicity including VLS which limits its therapeutic efficacy [1], [2], [3], [4], [5]. In the current study, we first evaluated whether resveratrol plays any role in the prevention of HDIL-2-induced VLS. For this purpose, we induced VLS in normal mice with HDIL-2. Resveratrol was given to mice one day before HDIL-2 treatment and once a day during the treatment until the day of sacrifice of mice. Four sets of experiments were established: IL-2 treatment (IL), resveratrol treatment (Res), IL-2 + resveratrol co-treatment (IL+R), and PBS treatment (PBS). The results showed that the vascular leak was dramatically suppressed by resveratrol treatment. Serum levels of TNF-α were also significantly decreased in resveratrol treatment group (Fig. 1). These results demonstrated that resveratrol can prevent IL-2-induced VLS.

Next, we tested whether resveratrol exerts a similar effect in melanoma-bearing mice. To this end, C57BL/6 mice were injected *i.v.* with B16F10 melanoma cells (5×10^5^ cells/mouse). On day 9, mice were treated with IL-2 or resveratrol as described above. The mice were euthanized for evaluation of VLS on day 12. Our results showed that resveratrol significantly inhibited VLS in tumor-bearing mice both in the lungs and liver to a similar extent when compared to that seen in the non-tumor-bearing mice (Fig. 2A and 2B). The metastasis and tumor growth in lungs (seen as black nodules) were significantly inhibited by IL-2 or IL2+resveratrol co-treatment. Moreover, IL2+resveratrol co-treatment was more effective in inhibiting tumor metastasis than IL-2 treatment alone (Fig. 2C–2E). These results indicated that resveratrol can ameliorate IL-2 toxicity while promoting the effectiveness of IL-2 in metastatic melanoma therapy.

### Resveratrol does not affect infiltration of inflammatory cells but protects endothelial cell integrity and prevents the cells from undergoing apoptosis

We previously found that HDIL-2-therapy caused endothelial cell (EC) damage by activating LAK cells [26], [28], [29]. Therefore, we investigated if resveratrol-mediated suppression of VLS resulted from its protective effect on ECs by pursuing ultrastructural studies of the lungs. As shown in Fig. 3, ECs from PBS or resveratrol group showed normal morphological features. In contrast, ECs from IL-2-treatment group revealed extensive damage, including cell membrane folding or break and vacuolation of cytoplasmic particles. Some of the endothelial cells were severely damaged, with only extended cell membrane remnants remaining. Cellular debris from ECs was found in the blood capillary lumen [26]. Interestingly, IL2+resveratrol treatment exhibited morphologically normal ECs and the basal lamina, whereas we only found minor damage in few endothelial cells.

We wondered whether suppression of IL-2 induced VLS by resveratrol resulted from inability of inflammatory cells to infiltrate into the organs. Therefore, the inflammatory cell extravasation was examined under microscope. The results showed that the IL-2 treatment induced significant perivascular infiltration both in lungs and liver. Notably, IL-2 + resveratrol co-treatment also exhibited similar levels of perivascular infiltration as the IL-2 treatment. The degree of infiltration was also measured by counting the layers of cell infiltration around each vessel and averaging the numbers for each group (Fig. 4). These data suggested that the suppression of VLS seen in IL-2 + resveratrol treated-mice was not due to the inability of infiltration of inflammatory cells.

We previously reported that apoptosis could be one of the mechanisms of EC damage [26]. Hence, we applied TUNEL assay to examine apoptosis in lung sections. Our results showed that IL-2-treated mice exhibited a large number of ECs that had undergone apoptosis, which was determined by TUNEL-positive staining. In contrast, IL-2+resveratrol treated-mice did not show TUNEL-positive cells (Fig. 5). These data suggested that resveratrol can prevent VLS by protecting ECs from ultrastructural damage and apoptosis.

### Resveratrol induced expansion and suppressive functionality of myeloid-derived suppressor cells (MDSC)

To explore the underneath mechanisms that contribute to the suppressive function of resveratrol on VLS, we studied the contribution of the suppressor cells in the inhibition of VLS by resveratrol. To this end, we focused on studies of Treg and MDSC. Our previous study showed that IL-2 treatment could induce expansion of CD4^+^FoxP3^+^ Treg; these Treg can suppress the cytolytic killing of ECs by LAK cells *in vitro* [12]. In the current study, we measured the percentage of Treg in spleen, lung and liver. On day 12, the splenocytes and the infiltrating-mononuclear cells from lung and liver were prepared for staining of CD4 and FoxP3. The results showed that IL-2 caused an expansion of Treg in spleen, lung and liver. However, resveratrol treatment did not induce their expansion; instead, it inhibited Treg expansion to some extent (Fig. 6). Simultaneously, we stained cells for Gr-1 and CD11b expression. The results showed that either resveratrol or IL-2 could induce the expansion of MDSC. Moreover, combination of IL-2+resveratrol caused a further induction of MDSC (Fig. 7). These results suggested the possibility that MDSC rather than Treg probably contributed to the suppression of VLS in resveratrol treatment.

To address this further, we examined the suppressive ability of MDSC on LAK cytotoxicity and proliferation. To this end, LAK cells from IL-2-treated mice were isolated, and then incubated with MDSC at 10∶1 ratio (LAK: MDSC) for 2 h at 37°C. For the cytotoxicity assay, the mixture of the culture was added to ^51^Cr-labeled EC target (TME cell line) at 50∶1 ratio (E:T) followed by 4 h ^51^Cr-release assay. For the proliferation assay, the mixture of the culture was supplemented with ^3^H-thymidine and continued culture for 16 h and then the ^3^H-thymidine incorporation was measured. To our surprise, the results showed that MDSC from IL-2+resveratrol group (named as R-MDSC) significantly inhibited LAK cytotoxicity and proliferation; however, MDSC from IL-2 group (named as IL-MDSC) did not show any suppressive effects on either LAK cytotoxicity or proliferation (Fig. 8A & 8B).

To further evaluate whether these MDSCs play a suppressive role in the development of VLS *in vivo*, we transferred these MDSCs intravenously into naïve mice and induced VLS in the recipient mice on the same day of the cell transfer. The results showed that R-MDSC caused marked inhibition of VLS; again, IL-MDSC did not inhibit VLS at all (Fig. 8C). Correspondently, we found that resveratrol induced the expression of Arg1 in MDSC but IL-2 did not. Interestingly, IL-2 and resveratrol co-treatment induced the most expression of Arg1 (Fig. 8D). This could be because resveratrol is more effective in activated cells than unactivated cells [30]. We also found that R-MDSC and IL-MDSC have different expression level of AhR, which is the main receptor for resveratrol-derived signaling [23]. We found that resveratrol increased the expression of AhR in MDSC but IL-2 did not. Similar to the expression of Arg 1, IL-2 and resveratrol co-treatment induced the most expression of AhR (Fig. 8E). All together, our results indicated that resveratrol can elicit or enhance the suppressive functionality of MDSC that could contribute to the suppression of VLS.

### Resveratrol induced expression of FoxO1 in tumor cells

Thus far, we demonstrated that resveratrol can ameliorate IL-2 toxicity while promoting the effectiveness of IL-2 in metastatic melanoma therapy. We showed that resveratrol-treated MDSC could contribute to the suppression of the cytolytic activity of LAK cells on ECs. However, MDSC are generally recognized for their role in helping evasion of tumor by suppressing the host immunity in situ of the tumor. Our results raised the possibility that resveratrol possesses the specific characteristic that can overcome the suppressive effect of MDSC to the tumor. To explore the possible mechanisms, we studied whether resveratrol played a role in the expression of the tumor suppressor genes by analysis of FoxO1 gene expression. To this end, we resected the primary melanoma in the skin and examined the transcripts of FoxO1 by QPCR. The results showed that either resveratrol or IL-2 treatment can induce the expression of FoxO1 in the tumor *in vivo*. IL-2 and resveratrol co-treatment induced the highest expression of FoxO1 (Fig. 9A). Correspondently, the growth of the tumor was significantly inhibited by the treatment which was demonstrated by the decreased tumor weight while IL-2 and resveratrol co-treatment can exert the maximal inhibitory effects on the tumor growth (Fig. 9B).

We also cultured B16F10 cells with IL-2 and/or different dose of resveratrol. We found that resveratrol could induce the expression of FoxO1 in a dose-dependant manner. However, IL-2 alone did not induce the expression of FoxO1. This concentration of IL-2 is effective in LAK cell development [26]. With IL-2 and resveratrol co-treatment, the expression levels of FoxO1 were not different from the resveratrol treatment alone (Fig. 9C), which suggest that IL-2 does not directly affect the expression of FoxO1 in B16F10 tumor cells. However, in the *in vivo* experiment, HDIL-2 administration did induce the expression of FoxO1 in the primary tumors (Fig. 9A). These results indicate that the release of other factors from the HDIL-2-induced inflammation could trigger the expression of FoxO1 in the tumor, and such factors work together with resveratrol co-treatment to further enhance the expression of FoxO1.

### Resveratrol makes melanoma more susceptible to the cytotoxicity of LAK cells by increasing the sensitivity of the tumor cells to the cytotoxicity

It has been shown that resveratrol can suppress melanoma growth by inducing the cell-cycle interruption and apoptosis in the tumor cells; resveratrol also can enhance the chemical cytotoxicity to the tumor by the chemotherapeutic agents [24], [25]. In this study, we studied whether resveratrol could affect the LAK cytotoxicity to the tumor. To this end, LAK cells were used as the effectors and generated *in vitro* from splenocytes of normal mice as described in the *Materials and Methods*. B16F10 cells were used as the targets. We pre-incubated the targets with resveratrol at 25 µM for 2 h at 37°C before the 4 h cytotoxicity assay. The results showed that the pre-treatment of resveratrol significantly increased the number of the killed targets by LAK cells (Fig. 9D).

The above results suggested that resveratrol can directly induce the expression of the tumor suppressor gene FoxO1, thereby leading to the suppression of the tumor growth and cell death by triggering autophagy [31], [32]. IL-2 can facilitate such effects by the unknown factors from the HDIL-2-induced inflammation. Meanwhile, resveratrol directly enhances the susceptibility of B16F10 tumor cells to the LAK cytotoxicity. Together, these mechanisms are able to overcome the suppressive activity of MDSC against the immunity of the host that eventually leads to the tumor regression.

## Discussion

Our results support a potential use of resveratrol in HDIL-2 treatment against melanoma and revealed some mechanisms. The resveratrol treatment effectively inhibited the development of VLS that is the most severe side-effect from HDIL-2 therapy. Meanwhile, the co-treatment with resveratrol and HDIL-2 promoted the efficacy of the tumor therapy arisen from either resveratrol or HDIL-2 treatment. Resveratrol played a differential role in the protection of EC injury and killing of the tumor. On the one hand, resveratrol prevented ECs from the ultrastructural damage and apoptosis; on the other hand, it increased the tumor killing by enhancing the susceptibility of the tumor to the cytotoxicity killer cells.

To understand how resveratrol played the suppressive role in the development of VLS, we focused on the study of Treg and MDSC, the two main populations of the immune suppressors. We found that resveratrol induced the expansion of MDSCs that can suppress the cytolytic killing of ECs and the development of VLS while the number of Treg decreased at the same time. Previous studies from our lab and the others also showed that resveratrol inhibited the production of TGF-β and the expansion of Treg in the tumor-bearing mice while it induced the expansion of MDSCs that suppressed the development of chronic colits [33], [34]. These results demonstrate that MDSC is the predominant suppressor cells than Treg in the development of VLS. It could be true since MDSCs are the dominant suppressor cells that induce the tumor escape than Treg [35]. This conclusion could be corroborated by the fact that HDIL-2 treatment can induce the expansion of Tregs but they are not able to prevent the development of VLS [12].

There are conflicting reports on whether MDSC are involved in the induction of Treg [13]. It was also reported that both Treg and MDSC could expand in the tumor-bearing mice, and that the expansion of the two populations were not related [36]. Here, we showed that IL-2 treatment induced both MDSC and Treg expansion. However, resveratrol treatment only induced MDSC expansion while it inhibited Treg expansion. Therefore, our current study demonstrates a differential role of resveratrol on Treg and MDSC.

The expansion of MDSC in HDIL-2 immunotherapy was found in patients with renal cell carcinoma (RCC) therapy. In these patients, an increased secretion of Arg1 in the patients blood was also detected [37], [38]. However, the role of MDSC in the development of HDIL-2-induced VLS and their functionality has not been studied before. In the current report, we found that HDIL-2-induced MDSCs from spleen were not suppressive. This is not surprising considering that MDSCs from spleen of naïve mice or tumor-bearing mice do not possess the suppressive function; but they become suppressive after culture with tumor-derived factors, which is corroborated by the fact that MDSC from tumor tissue rather than spleen possess the suppressive characteristics. In these studies, the authors claimed that the regaining of suppressive function was because these MDSC differentiated into suppressive macrophages (F4/80^+^) [35], [39], [40]. In our report, resveratrol administration recovered their suppressive functionalities including the expression of Arg1, the suppression of the proliferation and cytotoxicity of LAK cells, as well as suppression of the development of VLS after the adoptive transfer (Fig. 8A–8D).

Whether resveratrol can induce the differentiation of the suppressive macrophages or how resveratrol recoveres the suppressive function of MDSC is not clear. We examined the interaction between resveratrol and MDSC. AhR functions as one the main receptors for resveratrol [23]. Our results demonstrated that resveratrol could induce the expression of AhR in the MDSC while IL-2 could not. Interestingly, IL-2 and resveratrol co-treatment induced the most expression of AhR (Fig. 8E). This could be because resveratrol is more effective in activated cells than unactivated cells [30]. We noted that the expression of AhR was positively related to the expression level of Arg1 and suppression functionality of MDSC. IL-MDSC showed the lowest level of AhR whereas R-MDSC showed the highest level of AhR. We assume that the signals from AhR may be critical for MDSCs to recover their function. The signals from AhR-resveratrol reaction might act as a mimic to the tumor-derived signals that induce the suppressive function of MDSCs. Further studies are needed to clarify this hypothesis.

Resveratrol has been shown to possess chemopreventive activities such as suppression of tumor cell proliferation, induction of tumor cell apoptosis, and enhancement of chemosensitization of tumor cells [21], [22], [24], [25]. It is known that resveratrol can directly induce cell-cycle disruption and apoptosis in chemoresistant B16 melanoma that leads to tumor regresson; meanwhile, resveratrol makes melanomas more susceptible to the chemotherapeutic drugs [24], [25]. Our results showed: (1) resveratrol significantly enhanced the expression of FoxO1 gene of melanoma (Fig. 9A-9C). (2) resveratrol dramatically increased the susceptibility of melanoma to LAK cytotoxicity (Fig. 9D). It is known that FoxO1 controls the tumor growth; the expression of FoxO1 inhibits the tumor growth and triggers the ultimate death of tumor [31], [32]. We propose that the above two effects of resveratrol might compensate and overcome the suppression of MDSC on the tumor infiltrating T lymphocytes so as to promote anti-tumor immunity.

The response to immunotherapy with HDIL-2 in patients with metastatic RCC and melanoma is about 15% to 23%. The immune suppression caused by the HDIL-2 treatment, such as induction of Tregs, could be part of the reason why HDIL-2 remains less effective [1], [2], [3], [4], [5], [37], [38]. Our results indicate that induction of MDSC is also an important factor in affecting the results of the treatment. The functional status of immune suppression of MDSC could be related to the responsiveness of the therapy in patients. The clinical evidence is needed to further elucidate such an issue. Resveratrol could be act in two beneficial ways in HDIL-2 therapy by suppressing endothelial damage while enhancing sensitivity of tumor killing. In our previous studies, we have shown that melanoma cells and ECs can show differential susceptibility to LAK lysis based on the expression of CD44 variant isoforms [27]. Therefore, resveratrol might induce the differential expression of CD44 variants in melanoma cells, ECs, LAK, Treg, and MDSCs that contributes to the differential role of resveratrol and MDSC in EC protection and tumor killing. Further studies are necessary to address the role of resveratrol in modulating CD44 isoform expression.
